# Prevalence of *Escherichia coli* O157:H7 and associated factors in under-five children in Eastern Ethiopia

**DOI:** 10.1371/journal.pone.0246024

**Published:** 2021-01-28

**Authors:** Dawit Kassaye Getaneh, Lemessa Oljira Hordofa, Desalegn Admassu Ayana, Tesfaye Sisay Tessema, Lemma Demissie Regassa

**Affiliations:** 1 College of Veterinary Medicine, Haramaya University, Dire Dawa, Ethiopia; 2 College of Health and Medical Sciences, Haramaya University, Dire Dawa, Ethiopia; 3 Institute of Biotechnology, Addis Ababa University, Addis Ababa, Ethiopia; University of Texas at San Antonio, UNITED STATES

## Abstract

**Background:**

*Escherichia coli* O157:H7 (*E*. *coli* O157:H7) is one of the most potent zoonotic pathogens that causes mild diarrhea and leads to hemolytic uremic syndrome or death. This study was aimed to assess the prevalence and determinants of *E*. *coli* O157:H7 related to diarrhea among under-five children with acute diarrhea.

**Methods:**

A cross-sectional study design was carried out in 2018 on 378 under-five-year children recruited randomly from hospitals in Eastern Ethiopia. Stool specimens were collected and processed using enrichment, differential and selective medium. Among isolates, *E*. *coli* O157:H7 was confirmed using latex test (Oxoid, Basingstoke, Hants, England). Factors associated with *E*. *coli* O157:H7 infection were identified using binary and multivariable logistic regression. Associations were reported by odds ratio with 95% confidence interval.

**Results:**

The prevalence of *E*. *coli* O157:H7 related diarrhea was 15.3% (95%CI: 11.8–19.5). The *E*. *coli* O157:H7 infection was positively associated with rural residence (AOR;3.75, 95%CI:1.26–11.20), consumption of undercooked meat (AOR;3.95, 95%CI: 1.23–12.67), raw vegetables and/or fruit juice (AOR;3.37, 95%CI:1.32–8.62), presence of bloody diarrhea (AOR;4.42, 95% CI:1.78–10.94), number of under-five children in a household (AOR;7.16, 95%CI: 2.90–17.70), presence of person with diarrhea in a household (AOR;4.22, 95% CI: 1.84–12.69), owning domestic animal (AOR;3.87, 95% CI: 1.48–10.12) and uneducated mother (AOR;3.14, 95%CI: 1.05–9.42).

**Conclusion:**

The Prevalence of *E*. *coli* O157:H7 related diarrhea among under-five children is relatively high in Eastern Ethiopia. The E. coli infection was associated with sanitation and hygiene in a household. Thus, education focused on food cooking and handling, child care, and household sanitation associated with animal manure in rural resident children are helpful in.

## Introduction

Diarrheal disease is the second leading cause of mortality and morbidity among under-five (U5) children in the developing world. It is responsible for the death of more than 1,400 children every day, and 88% of deaths are concentrated in South East Asia and sub-Saharan Africa [1]. In the past five years, community-based prevalence studies in different regions of Ethiopia have indicated the prevalence of diarrheal disease was ranged from 8% to 36% [2–4].

Although *E*. *coli* is a ubiquitous intestinal bacterial flora of animals and humans, there are several pathogenic stains associated with diarrhea, collectively called diarrheagenic *E*. *coli*, namely; enterohaemorrhagic *E*. *coli (*EHEC*)*, enterotoxigenic *E*. *coli*, enteropathogenic *E*. *coli*, enteroaggregative *E*. *coli*, enteroinvasive *E*. *coli*, and diffusely adherent *E*. *coli* [5]. The EHEC serotype *E*. *coli* O157:H7 produces a powerful toxin responsible for the severe cause of hemolytic uremic syndrome, end-stage renal failure, and even death in humans [6]. *E*. *coli* O157:H7 is responsible for 20% of foodborne outbreaks globally [7]. It is predominantly associated with childhood diarrhea with an isolate rate of 39.3% in Khuzestan province of Iran [8], 33.6% in Norway [9], and up to more than 20% in Nigeria [10, 11] from community-based prevalence studies.

Although there is a paucity of epidemiological information among children in Ethiopian, a prevalence of 14% was reported in a Hospital-based cross-sectional study at Bahir-Dar [12]. In low resource countries like Ethiopia, where childhood diarrheal disease is a common, significant unsanitary condition with livestock product consumption habits, close relationship with reservoir animals, and poor health infrastructure, there is no comprehensive information about the epidemiology of *E*. *coli* O157: H7. Hence, this study aims to determine the prevalence of *E*. *coli* O157: H7 and its associated factors among under-five children in Eastern Ethiopia.

## Materials and methods

### Study area and period

The study was carried out in Hospitals found in Eastern Ethiopia, namely, Haramaya District Hospital (HDH) and Hiwot-Fana Specialized University Hospital (HFSUH) from January to March 2018. Haramaya District Hospital is a primary hospital located at approximately 509 km East of Addis Ababa, the capital City of Ethiopia, and 19 Km ahead to reach Harar. Geographically, this area lies between 9^0^26’N latitude and 42^0^ 01’E longitude, at an altitude of 2047 meters above sea level. HFSUH, the largest government Hospital in Eastern Ethiopia, is found in Harar town, the capital City of Harari region. The region has a total population of 183,344, of which 92,258 were males and 91,086 females, with 99,321 (54.17%) of the population are urban inhabitants [13]. Geographically, the region is situated between Latitude: 9°18′49″ N and Longitude of 42°07′05″ E at an elevation of 1917 m above sea level. The area has a mean temperature ranging from 10 to 18 ^O^C with a relative humidity of 65%. It receives an average annual rainfall of 800 mm with a bimodal distribution of the seasonal pattern, peaking in mid-April and mid-August of the year; however, there is a variation from year to year.

### Study design

A Hospital-based cross-sectional study design was used to determine the prevalence of *E*. *coli* O157:H7 and its associated factors among children with acute diarrhea.

### Population

All under-five children with acute diarrhea who visited HDH and HFSUH were considered the source populations. From the source population, those who selected to participate in the study were considered the study population.

### Inclusion and exclusion criteria

All under-five children with acute diarrhea who visited HDH and HFSUH during the study period were included. Those on antibiotics at the preceding week and the study period were excluded not to hide the etiology of diarrhea.

### Sampling methods and procedure

**Fig 1:**
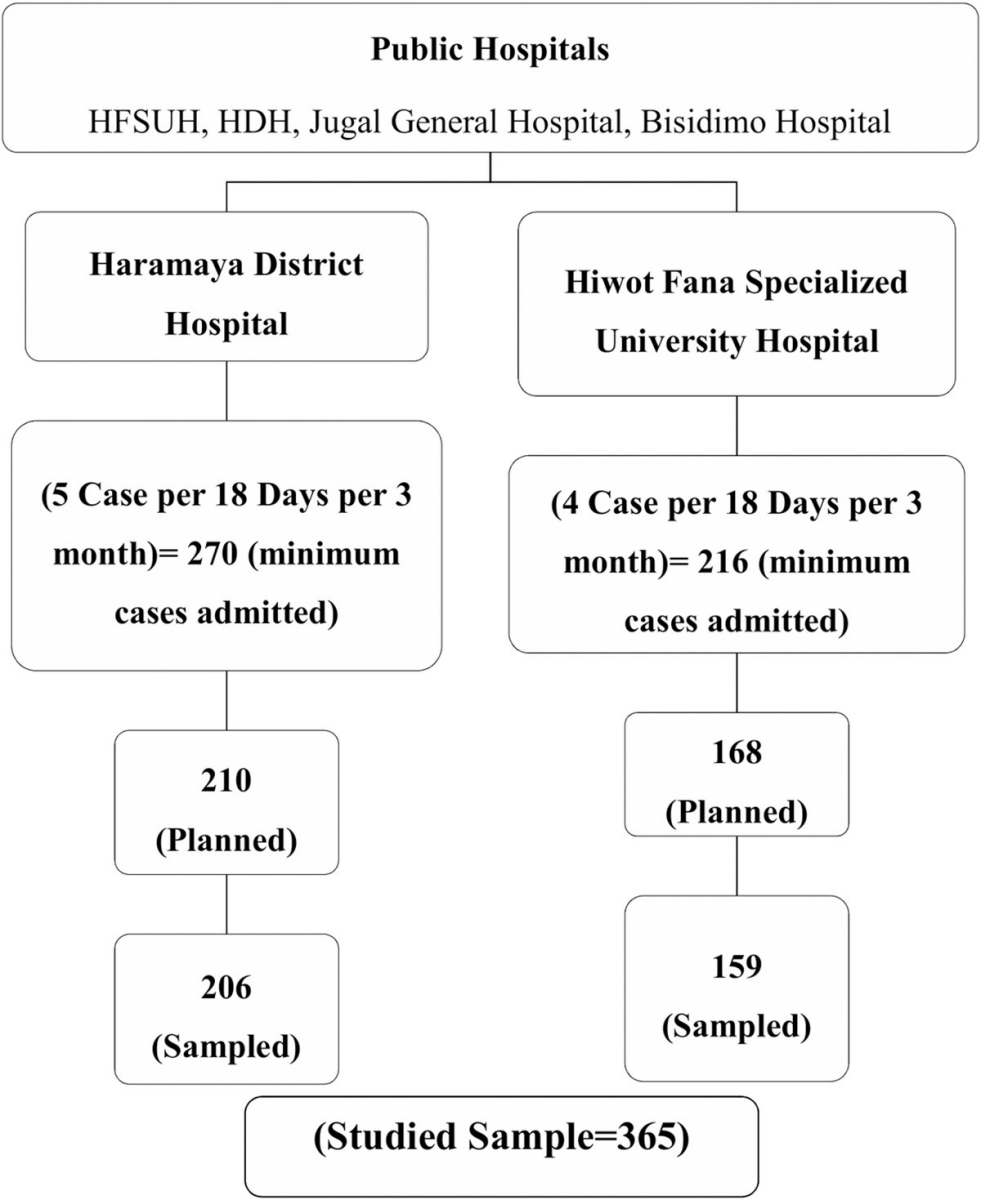
Schematic representation of the sampling procedure for selecting the study population from hospitals, Eastern Ethiopia, 2018.

The sample size was determined based on the previous study findings [14] following a single and double proportion formula for the prevalence of *E*. *coli* O157: H7 and the associated factors, respectively. As a result, 378 under-five-year children were planned to include. From the four (HFSUH, HDH, Jugal General Hospital, and Bisidimo Hospital) Public Hospitals found in the study area, two Hospitals (HFSUH and HDH) were selected randomly by lottery method. The sample size was proportionally allotted based on their patient case flow. A three-month (September, October, and November) mean diarrhea cases were estimated from the registration books of under-five children to guess the probable case flow on each hospital per day. Then the study subjects were recruited consecutively until the desired sample was reached (Fig 1).

### Data collection and stool sample collection

After written informed voluntary assent was obtained, a structured pretested questionnaire was used and completed by two trained laboratory technicians in a face-to-face interview basis. Participants were also asked and guided by the laboratory technicians to give the stool sample. On-spot gross examination of stool samples was performed to note the type of diarrhea at the collection time. Swabs with visible stool staining were prepared and collected in a sterile hi-culture test tube with Buffered Peptone Water (BPW) (Oxoid Ltd., Hampshire, England**)** and labelled. Lastly, the sample was transported in an icebox within 24 hours of collection to the Haramaya University laboratory and stored at refrigeration temperature (+4 ^o^C). However, all samples were processed by well-experienced laboratory experts within two days of sample collection.

### Laboratory processing: Isolation and identification of *E*. *coli* O157:H7 *(S1 File)*

The sample was enriched by Modified tryptone soy broth supplemented with Novobiocin (mTSB+N) at the ratio of 1:9 BPW to mTSB+N and incubated overnight at 41.5±0.5°C (24 hrs.). All media required for the isolation and biochemical characterization of *E*. *coli* were prepared as per the manufacturer’s instructions. The enriched sample was cultured aerobically at 37°C for 24 hours on MacConkey Agar (IVD, UK) (for easy identification of lactose fermenting organisms) and Eosin Methylene Blue Agar (Oxoid LTD, Basingstoke, England), for easy identification of the green metallic sheen appearance characteristic of *E*. *coli* colonies. Colony morphology and reaction on agar media were noticed and registered. Biochemical tests were performed on presumptive *E*. *coli* colonies using the standard bacteriological procedures described by Quinn *et al*. [15]. Isolates with biochemical test results for indole, methyl red, Voges-Proskauer, and citrate utilization (IMViC) (+ +—-), respectively, were presumed as *E*. *coli* isolates (SI). Furthermore, chromogenic Rainbow Agar O157 (Biolog, Hayward, HUSA) and Sorbitol MacConkey containing cefixime and tellurite (CT-SMAC) (Oxoid, England) media were used to notice the typical *E*. *coli* O157:H7 colonies after overnight culture (24hrs) at 37°C. *E*. *coli* isolates that appeared pink on Rainbow O157 and non-sorbitol fermenting on CT-SMAC were presumed as presumptively *E*. *coli* O157:H7 colonies [16, 17]. Then confirmation of *E*. *coli* O157:H7 was performed using *E*. *coli* O157:H7 latex test (Oxoid, Basingstoke, Hants, England). A drop of latex was dispensed near the edge of the circle on the reaction card. Using a loop, up to 10 separate presumptive colonies were emulsified in a drop of 0.85% saline solution to increase the probability of detecting *E*. *coli* O157:H7. Latex quality control was performed before the samples were tested using control latex. Agglutination of the latex within a minute was registered as a positive result (S1 File).

### Study variables

#### Dependent/outcome variables

*E*. *coli* O157:H7 related diarrhea.

#### Independent/explanatory variables

The explanatory variables included were the following:

- Socio-demographic, behavioral, and environment-related factors concerning the study participants (under-five-year children) include; age, residence, sex, sharing a home with animals, animal manure contact, breastfeeding status, type of diarrhea, and food consumption habits.
- Socio-demographic, behavioral, and environmental factors related to the households of the child includes; age of the mother, marital status of the mother, total family size, diarrhea in the household other than the sampled child, number of under-five children, hand washing habits, child food preparing condition, drinking water treatment method and per capita water consumption.

### Data quality control

The questionnaire was pretested on a 5% sample for one-week period. Two-day training was given to laboratory technicians to ensure their understanding of the data collection technique. Supervision was made daily and the filled questioner is collected back by checking its completeness on a daily base. During laboratory analysis, calibrated equipment and standard operating procedures were used. Culture media were prepared and sterilized based on the manufacturer’s instructions. All dispensed culture media were checked for sterility before culturing the samples after 37°C overnight incubation. Appropriate standard strain, in this case, *E*. *coli* O157:H7 (ATCC 43894) was used as a positive control, and Biosafety cabinet level II was used during each stool culture and culture transfer. Double data entry was conducted and identified errors were corrected after revising the original questionnaire and recording formats.

### Data analysis

The data were coded, entered, and cleaned using EpiData version 4.2. Then the data were exported into STATA version 14 for processing and analysis. Descriptive statistics: mean, standard deviation, and proportion were used to present the findings. Binary logistic regression was conducted and variables with p≤0.25 were selected as a candidate to be included in the multivariable logistic regression analysis in an attempt to control for potential confounding variables, and the adjusted odds ratio was determined. Collinearity between variables was also checked by the standard error, and Hosmer and Lemshow test assured the model fitness. Throughout the data presentation, a p-value of less than 0.05 was considered statistically significant.

### Ethical statement

We obtained ethical clearance from the Institutional Health Research Ethics Review Committee (IHRERC) of Haramaya University. Informed, voluntary, written, and signed consent was obtained from the mother/caregivers of the children and the head of each hospital after the aim of the study, and the nature of the study is fully explained to them. Parents/guardians of the child were also informed about the confidentiality of the information obtained and about their full right to refuse or drop participating in the research. We covered the cost of investigation and treatment of confirmed cases for those who could not afford to pay. Besides, we assured them to cover the cost associated with confirmed cases if they are unable to afford the payment. Since the result of the laboratory took about weeks, the address of the participants, mobile phone number of the family, and in those who did not have a mobile phone, their relative address of the family was documented. Until the laboratory report, the physician on duty treated all children based on the existing guideline. We communicated the result of the participants to their parents/ guardians immediately. We tried to reach all families of the child through the health extension workers of their respective Kebeles taking into account the consequences of the case if not managed properly.

## Results

### Sociodemographic characteristics

Data was collected from 365 (96.6% response rate), 206(56.4%) from HDH, and 159 (43.6) were from HFSUH. Of the total, 191 (52.33%) were males. The mean age of children was 18.7 months (SD±14.5) and 28.94 (SD±5.68) for mothers. Most of the children’s mothers were married (81.94%) and 70.7% rural residents. More than half (257/365) had no formal education, and 79.45% (290/365) were housewives. Two hundred twenty-one (73.67%) Children’s’ fathers had no formal education (Table 1).

**Table 1:** Characteristics of the sample (children) and their parents, Eastern Ethiopia, 2018.

Variables	Category	Frequency (%)
**Child Age (months)**	≤12months	197 (53.97%)
	>12months	168 (46.03)
**Child sex**	Male	191 (52.33)
	Female	174 (47.67)
**Maternal age (years)**	15-24yrs	108 (30.17)
	25-34yrs	161 (44.97)
	35–4	89 (24.86)
**Residence**	Rural	258 (70.68)
	Urban	107 (29.32)
**Maternal educational level**	No formal	257 (70.41)
	Formal	108 (29.59)
**Father’s educational level**	No formal	221(73.67)
	Formal	79 (26.33)
**Mother occupation**	House wife	290 (79.45)
	Merchant	48 (13.15)
	Farmer	14 (3.84)
	Government	13 (3.56)
**Family size**	≤4	193 (52.88)
	>4	172 (47.12)
**Number of U5 children in HH**	0–1	224 (62.22)
	2–3	136 (37.78)
**Exclusive breast feeding**	Yes	52 (14.40)
	No	309 (85.60)
**Persons with diarrhea in the house**	No	239 (65.48)
	Yes	126 (34.52)

Notes: U5, under five years old, HH; household.

Regarding the household and environmental characteristics, 40% (146/365) of households have only one room, 81 (36.49) sharing room with animals. More than half households (253/365) had traditional latrines and 11.78% use open field as a latrine. The majority of the households (62.47%) get water from taps while 9% use surface water. Overall, 22.74% eat undercooked meat, 23.20% uncooked vegetables, 12.60% drink raw milk, and 22.47% fruit juice (Table 2). Household, environmental and behavioral characteristics are described in Table 2.

**Table 2:** Household, environmental and behavioral characteristics of study participants, Eastern Ethiopia, 2018.

Variables	Category	Frequency (%)
**Number of rooms in HH**	One	146 (40.00)
	Two	150 (41.10)
	More than two	69 (18.90)
**Animal ownership**	No	143 (39.18)
	Yes	222 (60.82)
**Sharing a room with domestic animals (n = 222)**	No	141 (63.51)
	Yes	81 (36.49)
**Type of latrine**	Traditional	253 (69.32)
	Open field	43 (11.78)
	Public	36 (9.86)
	Ventilated improved pit	33 (9.04)
**Source of water**	Tap	228 (62.47)
	Well	104 (28.49)
	Surface	33 (9.04)
**Handwashing means**	Water only	206 (57.87)
	Water and ash	103 (28.93)
	Water and soap	47 (13.20)
**Household water treatment**	No	233 (64.36)
	Aqua tab	70 (19.34)
	Boiling	34 (9.39)
	Filtering	25 (6.91)
**Eating under cooked meat**	No	282 (77.26)
	Yes	83 (22.74)
**Drinking raw milk**	No	319 (87.40)
	Yes	46 (12.60)
**Eat raw vegetables**	No	278 (76.80)
	Yes	84 (23.20)
**Drink fruit juice**	No	283 (77.53)
	Yes	82 (22.47)

Note: HH; household.

### Prevalence and associated factors of *E*. *coli* O157:H7 related diarrhea

**Fig 2:**
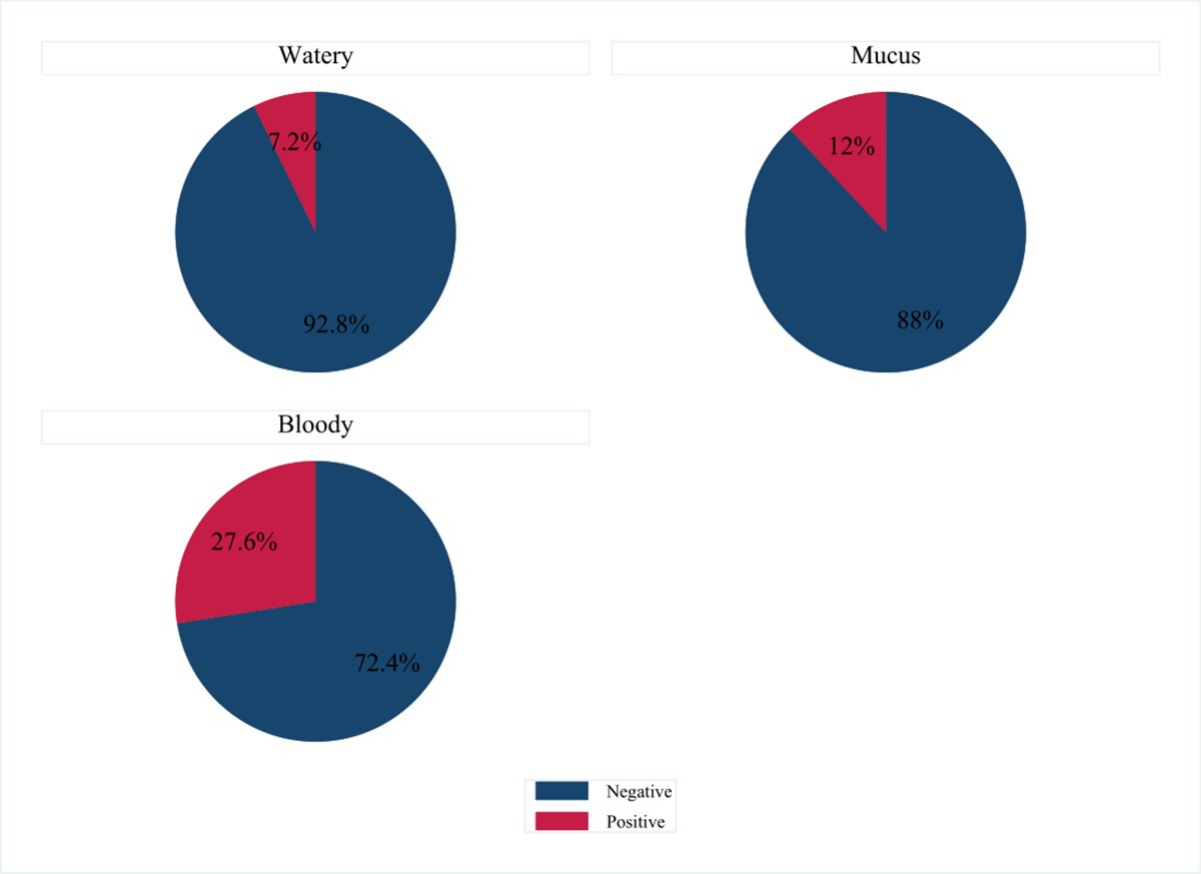
Prevalence of *E*. *coli* O157:H7 related diarrhea among under five aged children from Public Hospitals of Eastern Ethiopia, 2018.

Almost halve of the sample (181/365) was presented with watery diarrhea, while 13.7% with mucus and 36.7% dysentery. Upon laboratory investigation, *E*. *coli* O157:H7 strain was found in 56 children (15.3%; 95% CI: 11.8–19.4) children (Fig 2).

The prevalence of *E*. *coli* O157:H7 related diarrhea was significantly associated with rural residence, under-cocked meat consumption, raw vegetable consumption, dysentery, type of diarrhea, number of under-five children contained in the household, educational status of mothers, livestock ownership, and households with a history of diarrhea (Table 3).

**Table 3:** Multivariable analysis results of associated factors of *E*. *coli* O157:H7 among under-five children with diarrhea, Eastern Ethiopia, 2018.

Variables	Categories	COR (95%, CI)	AOR (95%, CI)
**Childs age**	>18	2.44 (1.37–4.35)	2.16 (0.78–5.97)
	≤18	1.00	1.00
**Residence**	Rural	2.83(1.29–6.21)	3.75(1.26–11.20) ***
	Urban	1.00	1.00
**Consume under cocked meat**	Yes	5.07(2.78–9.25)	3.95(1.23–12.67) ***
	No	1.00	1.00
**Raw milk consumption**	Yes	1.00	1.00
	No	0.39(0.18–0.89)	0.63(0.21–1.86)
**Consume Raw Vegetable**	Yes	2.99 (1.66–5.36)	3.37(1.32–8.62) **
	No	1.00	1.00
**Type of diarrhea**	Watery	1.00	1.00
	Bloody	3.94(2.04–7.62)	4.42(1.78–10.94) **
**Number of under-five children**	1–2 child	1.00	1.00
	3–4 children	4.44(2.41–8.17)	7.16(2.90–17.70) ***
**Mothers Education level**	No formal	1.87 (0.9–4.18)	3.14(1.05–9.42) **
	Formal	1.00	1.00
**Animal ownership**	Yes	2.70(1.37–5.31)	3.87(1.48–10.12)**
	No	1.00	1.00
**Diarrhea in Household**	Yes	3.64(2.02–6.57)	4.22(1.84–12.69) ***
	No	1.00	1.00
**Drinking water treatment method**	Boiling	1.00	1.00
	Aqua tab	1.78 (0.35–9.06)	1.03(0.14–7.16)
	Straining	6.22(1.17–33.2)	6.8 (0.78–17.5)
	Nothing	3.32(0.76–14.4)	3.61(0.64–20.55)
**Hand washing ways usually practiced**	Water only	1.00	1.00
	Ash & water	0.36 (0.17–0.78)	1.32 (0.48–3.63)
	Soap & water	0.26(0.08–0.87)	0.29 (0.07–1.27)

**Notes:** COR; crude odds ratio, AOR; adjusted odds ratio, *; p-value<0.05

**; p-value<0.01

***; p-value<0.001.

Multivariable analysis showed the odds of *E*. *coli* O157:H7 related diarrhea among children from rural residence were 3.75 times (AOR:3.75; 95%CI:1.26, 11.20) higher than children from urban residential. The likelihood of *E*. *coli* O157:H7 related diarrhea is increased by about four-fold (AOR:3.95; 95%CI:1.23, 12.67) in children who consumed undercooked meat compared to those who did not eat under cooked meat. Children with the history of consuming raw vegetables were 3.37 (AOR:3.37; 95%CI:1.32, 8.62) times more likely to have *E*. *coli* O157:H7 related diarrhea than those without a history of consuming raw vegetables. Children with dysentery (bloody diarrhea) were 4.42 (AOR: 4.42; 95%CI:1.78, 10.94) times more likely to have *E*. *coli* O157:H7 related diarrhea than those with watery diarrhea. The likelihood of *E*. *coli* O157:H7 related diarrhea was seven times (AOR:7.16; 95%CI:2.90, 17.70) higher among children from a household with more than two children compared to a household with less than two children. Children from families who owned livestock were 3.87 (AOR: 3.87; 95%CI:1.48, 10.12) times more likely to have *E*. *Coli* O157:H7 related diarrhoea compared to children from families who did not have livestock. The odds of *E*. *coli* O157:H7 related diarrhoea were fourfold (AOR; 4.22; 95%CI:1.84, 12.69) greater among children from families with a recent history of diarrhoea compared to children from households without a history of diarrhoea.

## Discussion

This Hospital-based study provides results on the prevalence of *E*. *coli* O157:H7 and associated factors in under-five children with diarrhea in Eastern Ethiopia. The prevalence of *E*. *coli* O157:H7 among the children was 15.3% (95% CI: 11.8–19.4) and associated with being rural residents, under cocked and raw vegetable consumers, history of dysentery type of diarrhea, number of children, mothers uneducated, owned livestock, and history of diarrhea in the household.

The prevalence of *E*. *coli* O157:H7 infection was in agreement with the prevalence report of the study (14%) in Bahir-Dar town, Ethiopia [12] and it is comparable with the study (20%) in Benin City, Nigeria [10]. However, it is higher than the report of a study conducted in central Ethiopia in 2017 [18] and southern Ethiopia [19]. It is also much higher than studies conducted in other African countries [20, 21]. The discrepancy might be explained by the difference in sample size, source of study population difference, and method of sample collection.

Moreover, compared to hospitals-based studies of the same age group with diarrhea, the current prevalence was higher than studies conducted in some Asian countries, as the reported prevalence was 1.14% in Iran [22], and 4.61% in India [23, 24]. Compared to South American studies, in Argentina, the finding is also higher than the 10.1% report of Rivero *et al*. [25]. Generally, the relatively high prevalence of the organism obtained in the current finding might be because of the cosmopolitan and high level of animal-to human interaction in the study subjects than the above-mentioned countries, indicating *E*. *coli* O157:H7 to be an important diarrhea causing pathogen in the study population.

It was found that children from rural residents were about four times more likely to have *E*. *coli* O157:H7 compared to urban resident children. The finding is consistent with other studies conducted elsewhere [26, 27]. The positive association of the existence of high-density reservoir animals as a predictor of infection [28, 29] and the higher probability of rural residents to have animal contact with the predictor of rural residence for childhood diarrhea in this age group [30, 31] might support the finding.

Consuming undercooked meat and raw vegetables is significantly associated with the prevalence of *E*. *coli* O157:H7. This finding is in line with the report of previous studies [32–34]. This could be evidenced by the high level of animal manure contamination of vegetables through either irrigation by untreated surface water or animal manure usage as a fertilizer [35]. As indicated in this study, animal manure usage as a fertilizer was significantly associated with infection to the organism. The organism has been isolated from vegetable samples [36], which can survive outside the host reservoirs [37]. Although eating variety vegetables and fruits was reported as a protective factor [38], there were lettuce associated large outbreaks raised in Sweden in the year 2005 [39].

The likelihood of *E*. *coli* O157:H7 was more than four times higher among children with bloody diarrhea relative to those who experience watery diarrhea. Previous study reported was more likely to be isolated from visibly bloody stool than without visible blood and was the pathogen most commonly isolated from visibly bloody stool that yielded a bacterial enteric pathogen [33, 40, 41].

Meanwhile, the association of raw milk consumption and *E*. *coli* O157:H7 was not found statistically significant, although some authors described as consumption of raw milk is a risk factor for the occurrence of *E*. *coli* O157:H7 infection [42, 43]. However, the interpretation must be cautious because of the limitations posed by the small sample size (12.6%) of the children had consumed raw milk.

Age differences were not found independently associated with the occurrence of *E*. *coli* O157:H7. However, Kargar and Homayoon found that children (< 2) years) of age were at highest risk of infection with *E*. *coli* O157:H7 [44]. Since the data related to the exposure status of the child were not mentioned, it is difficult to justify. The contrary was reported as the odds of detection proportion of *E*. *coli* O157:H7 were high in those children above the mean age of more than 11%. The discrepancy seen with the current finding might be attributed to the older child (1–17 years) age group in the later study, which will influence the social and cultural behavior [45].

Children from households with livestock were four times more likely to contract *E*. *coli* O157:H7 than households without livestock, which is consistent with the report of a study on cattle owners [46] and a study conducted in Harare, Zimbabwe [47]. Since ruminants had been identified as the major reservoir of *E*. *coli* O157:H7, there could be a cross-infection to the children through either by direct live animal contact or with animal manure. A matched case-control targeted particularly in children under three years of age in Germany also indicates nine times the risk of developing infection in those who touched ruminants including goats and sheep [14]. In the meantime, studies reported that contact with farm animal manure or cattle and living in or visiting a place with farm animals be well-established risk factors for the occurrence of *E*. *coli* O157:H7 infection [32, 48].

Children whose mothers had no formal education were three times more likely to have *E*. *coli* O157:H7 than those with mothers with formal education. It is possible to suggest that mother’s education level might have a positive influence on preventive measures of diarrhea such as household hygiene and sanitation. Similarly, poor levels of household sanitation had been reported as a predictor of *E*. *coli* O157:H7 infection [38]. Evidences strength of the presence of low awareness on hygiene and sanitation among low-income mothers and this could expose a child to several cross-contaminations from one source to the other, indicative of poor personal hygiene [49].

Children of a household member with diarrhea prior to his/her diarrhea manifestation were four times more likely to contract *E*. *coli* O157:H7. The same finding was obtained by Rivas in their case-control study [38]. In addition, a child who was from a household having three to four under-five children had seven times higher odds of *E*. *coli* O157:H7 than a household with single to two children. Household under-five year number is a well-studied determinant factor associated with childhood diarrhea through its effect on overall household sanitation and hygiene levels [50–53]. Although the levels of contamination of the water sources are not well identified, drinking water treatment methods were not found associated with the occurrence of the organism. However, other researchers reported drinking contaminated water sources as a predictor for the occurrence of *E*. *coli* O157:H7 infection [36, 54–57].

However, the result obtained in this research work might be difficult to infer about the general source population because of the design. Besides, as study crosses sectional type, the absence and existence of the organism may not declare the absence of other enteric pathogens as a cause of diarrhea.

## Conclusions

The prevalence of *E*. *coli* O157:H7 among under-five children is found high. The likelihood of *E*. *coli* O157:H7 prevalence was increased among rural residents under cocked and raw vegetables, consumers who had dysentery, type of diarrhea, families with more than two children, uneducated mother, availability of livestock, and presence of family members with a history of diarrhea.

In line with the above conclusion, creating awareness on childcare and household sanitation is essential. Local health authorities should work in collaboration with the local agriculture and Livestock development bureau to take preventive hygienic measures associated with animal manure and the role of livestock’s and livestock products in the transmission pathway of *E*. *coli* O157:H7 to humans in all interfaces in rural residents. Finally, we recommend future research efforts must target at a community level the transmission chain of *E*. *coli* O157:H7 and its health impact in under five years.

## Supporting information

S1 File Laboratory SOP (Medias used, laboratory procedures, and antimicrobial disks used for their interpretation). (DOCX)

## List of abbreviations

BPW: Buffered Peptone Water
CT-SMAC: Cefixime Tellurite Supplemented Sorbitol Macconkey Agar
*E. coli*: *Escherichia coli*
EHEC: Enterohaemorrhagic *Escherichia coli*
HDH: Haramaya District Hospital (HDH)
HFSUH: Hiwot-Fana Specialized University Hospital
HH: House Hold
IHRERC: Institutional Health Research Ethics Review Committee
IMVIC: Indole, Methyl red, Voges-Proskauer, and citrate
mTSB+N: Modified tryptone soy broth supplemented with Novobiocin
U5: Under five
