# Supplementary material for: Prevalence of Escherichia coli O157:H7 and associated factors in under-five children in Eastern Ethiopia
Source: PLoS One. 2021 Jan 28;16(1):e0246024. doi: 10.1371/journal.pone.0246024 (PMC7842931; doi:10.1371/journal.pone.0246024)

1. **EMB (Eosin methylene blue) Agar** (Oxoid LTD, Basingstoke, England)

Direction:

- Suspend 37.5g of EMB agar powder in 1liter of distilled water
- Boil the suspension to dissolve completely
- Sterilize by autoclaving at 121oC for 15minutes
- Cool to 60oC and shake the medium in order to oxidize the Methylene blue
- Pour the suspension into sterile petredish
- Allow the medium to solidify and store at 4oC until inoculation
- Inoculate the faecal samples and incubated at 37°C for 24 hours

1. **Indole test**

1. Take a sterilized test tube containing 4 ml of tryptophan broth.

2. Inoculate the tube aseptically by taking the growth from 18 to 24 hrs culture.

3. Incubate the tube at 37°C for 24-28 hours.

4. Add 0.5 ml of Kovac’s reagent to the broth culture.

5. Observe for the presence or absence of ring.

Interpretation of Results

- Positive: Formation of a pink to red color in the reagent layer on top of the medium within seconds of adding the reagent.
- Negative: No color change even after the addition of appropriate reagent.

1. **Voges-proskauer (vp) test**
2. Prior to inoculation, allow medium to equilibrate to room temperature.
3. Using organisms taken from an 18-24 hour pure culture, lightly inoculate the medium.
4. Incubate aerobically at 35 oC for 24 hours.
5. Following 24 hours of incubation, aliquot 1ml of the broth to a clean test tube.
6. Re-incubate the remaining broth for an additional 24 hours.
7. To the aliquot (step 4 above), add 0.6ml of 5% alpha-naphthol. Next add 0.2ml of 40% KOH.
8. Gently shake the tube to expose the medium to atmospheric oxygen.
9. Allow the tube to remain undisturbed for 10-15 minutes.
10. Observe the medium for a pink-red color development. The test may be read for up to, but not beyond, one hour following addition of the reagents.

**Note:** If test reactions are negative (no red color produced) or questionable, the test can be repeated using the re-incubated broth (without reagents) from step 5 above. Re-incubation and repeat testing can be performed for up to 5 days.

Interpretation of Results

- A positive VP test is demonstrated by the development of a pink-red color on the surface of the medium 15 minutes to one hour after the addition of the reagents.
- A negative VP test is demonstrated by the appearance of a yellow color on the surface of the medium. Development of a copper-like color is also interpreted as negative.

1. **Methyl red (MR) test**

6a. Following 48 hours of incubation (step 5 above), aliquot 2.5 ml of the broth to a clean test tube

7a. Add five drops of methyl red indicator.

8a. Observe the medium for the immediate development of a red color.

Interpretation of Results

- A positive MR test is demonstrated by the development of a stable red color on the surface of the medium after the addition of methyl red indicator.
- A negative MR test is demonstrated by the development of a yellow color on the surface of the medium.

1. **Citrate utilization test:**
2. Inoculate Simmons Citrate Agar lightly on the slant by touching the tip of a needle to a colony that is 18 to 24 hours old.
3. Incubate at 35oC to 37oC for 18 to 24 hours. Some organisms may require up to 7 days of incubation due to their limited rate of growth on citrate medium.
4. Observe the development of blue color; denoting alkalinization.

Interpretation of Results

- - Citrate positive: Growth will be visible on the slant surface and the medium will be an intense Prussian blue. The alkaline carbonates and bicarbonates produced as by-products of citrate catabolism raise the pH of the medium to above 7.6, causing the bromothymol blue to change from the original green color to blue.
  - Citrate negative: Trace or no growth will be visible.  No color change will occur; the medium will remain the deep forest green color of the un-inoculated agar.  Only bacteria that can utilize citrate as the sole carbon and energy source will be able to grow on the Simmons citrate medium, thus a citrate-negative test culture will be virtually indistinguishable from un-inoculated slant

1. **Preparation of Buffered peptone water (Oxoid Ltd., Hampshire, England)**

Direction: add 20g of the powder to 1 liter of distilled water. Mix it well and sterilize by autoclaving at 121°C for 15 minutes.

Typical formula (g/l): peptone 10, sodium chloride 5. Final PH 7.2 ± 0.2

1. **Preparation of novobiocine and modified trypton soya broth (n+ mTSB) (Oxoid, England)**

Direction: Add 30 g of medium to 1 liter of distilled water. Mix it well and sterilize by autoclaving at 121°C for 15 minutes. Then add filter sterilized novobiocin additive after cooling to 50°C.

Typical formula g/l: Pancreatic digest of casein 17.0, enzyme digest of soybean meal 3.0, Sodium chloride 5.0, Di-potassium hydrogen phosphate 2.5, Glucose 2.5 g. Final PH~7.3 ± 0.2 at 25°C

1. **MacConkey agar (IVD, UK)**

Direction:- Disperse 52 g in 1 liter of distilled water. Soak for 10 minutes, mix and sterilize by autoclave at 121oC for 15 minutes. Cool to 47 ^o^C and mix well before pouring into Petri dishes and then dry the agar surface.

1. **Rainbow Agar O157 (BIOLOG, USA)**

A selective, chromogenic culture medium to aid in the isolation of verotoxin-producing strains of eschereshia coli, particularly serotypeO157:H7.

Storage: extremely hygroscopic. Store at 2-25^o^in a dry environment.

Direction: mix package contents in to 500ml of purified water. Heat gently to dissolve components and autoclave for 10 minutes at 15lbs. pressure and 121oc (medium is heat sensitive). Pour the medium in to petriplates, dispensing about 20ml per plate. The medium is ready to use as soon as it has cooled, gelled, and the surface has dried. The final medium should be transparent and virtually colourless.

1. **Preparation of CT-SMAC (Oxoid, England)**

Direction: Suspend 50.5g of the powder in 1 liter of distilled water. Heat with frequent agitation and boil for 1 minute to completely dissolve the powder. Autoclave at 121°C for 15 minutes and adds the following filter sterilized additives after cooling the medium to 50°C in water bath.

Potassium tellurite 2.5mg/l

Cefixime 0.05mg/l

Approximate formula per liter: peptone 20g, sorbitol 10g, bile salt 31.5g, sodium chloride 5g, neutral red 0.03, crystal violate 0.001g, agar 15g. Final PH 7.1 ± 0.2 at 25°C.

1. **Muller-Hinton agar preparation (Oxoid, England)**

Preparation: Suspend 38 g of the medium in one liter of purified water. Heat with frequent agitation and boil for one minute to completely dissolve the medium. Autoclave at 121°C for 15 minutes.

Formula (g/l): Beef Extract 2, Acid Hydrolysate of Casein 17.5, Starch 1.5, and Agar 17. Final.PH 7.3 ± 0.1 at 25°C

1. **Isolation and Identification of *E. coli O157:H7***


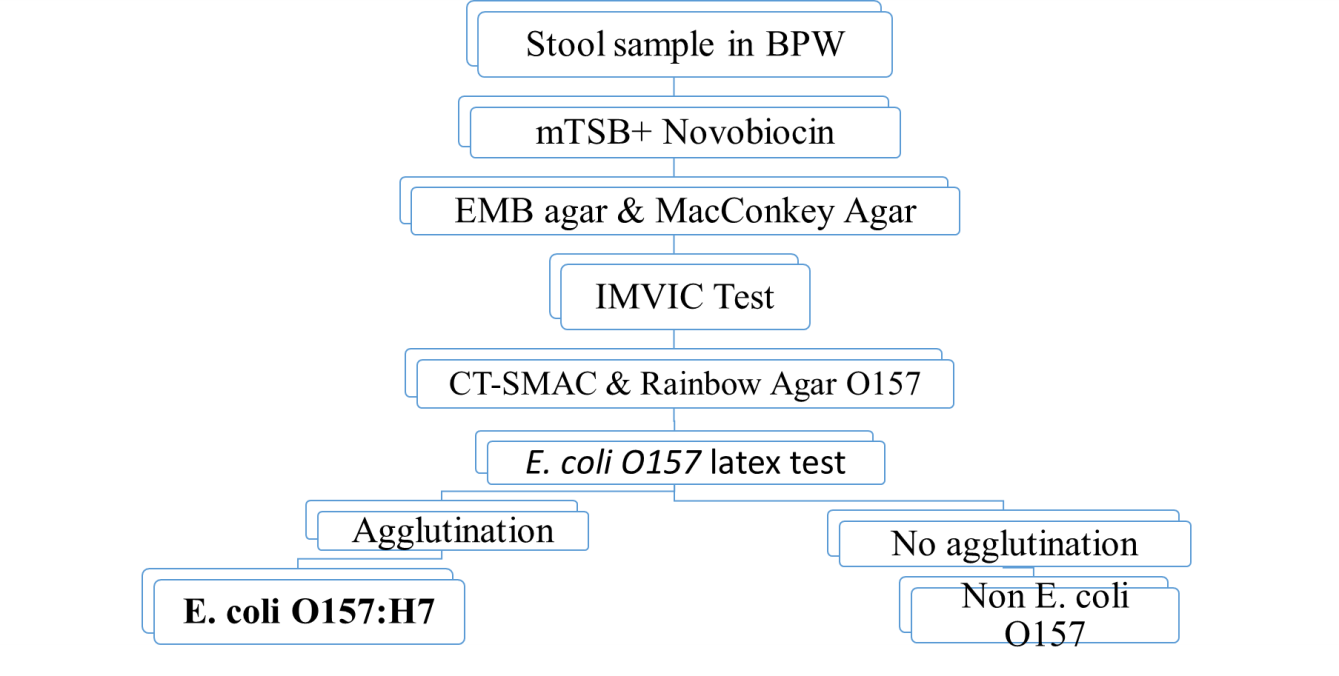

Supplement: S1 File — (DOCX) [file pone.0246024.s001.docx]
